# Exploring the benefits of a psychomotor intervention mediated by creative dance in community-dwelling older adults: development of new coordination and rhythm tests

**DOI:** 10.1186/s12889-025-21478-0

**Published:** 2025-05-14

**Authors:** Hugo Rosado, Patrícia Motta, Gabriela Almeida, Ana Cruz-Ferreira, Catarina Pereira

**Affiliations:** 1 Departamento de Desporto e Saúde, Escola de Saúde e Desenvolvimento Humano, Universidade de Évora, Largo Dos Colegiais 2, Évora, 7004-516 Portugal; 2 Comprehensive Health Research Centre (CHRC), Universidade de Évora, Évora, Portugal

**Keywords:** Aging, Dance, Coordination, Rhythm, Balance

## Abstract

**Background:**

Psychomotor intervention mediated by creative dance is emerging as an enjoyable practice that stimulates balance, coordination, and rhythm, benefiting physical function, cognition, and overall health in older adults. Despite the relevance of this practice, validated tests for assessing motor coordination and rhythm in older adults—essential parameters for motor control and movement regulation—are still needed. Thus, this study assessed the effects of a psychomotor intervention using creative dance in global motor coordination, rhythm, and balance in community dwellings. For this intent, one test to assess older adults' global motor coordination and one test to assess rhythm were developed and tested.

**Methods:**

This 12-week non-randomized clinical trial study included 38 participants (73.4 ± 5.7 years), allocated into two groups. The experimental group (EG; *n* = 19) attended the psychomotor intervention (3x/week; 50 min/session), while the control group (CG; *n* = 19) maintained their daily activities. Previous, global motor coordination and rhythm tests were developed based on the underlying literature and expert consultation. Their reliability and validity were determined. Fullerton Advances Balance Secale assessed Balance.

**Results:**

The test’s intra and inter-rater reliability was excellent, ranging 0.962–1.00. There were positive correlations between the test variables and theoretically-related parameters, *p* < 0.05. Within-group comparison revealed significant improvements after the intervention in the EG global motor coordination, rhythm, and balance, *p* < 0.05. Significant differences between groups concerning change (pre-post-intervention) in the previous variables were found, *p* < 0.05, with an effect size ranging 0.40–0.74.

**Conclusions:**

The current study supports the reliability and validity of the developed global motor coordination and rhythm tests in community dwellings. Nonetheless, further research is recommended to substantiate these findings in men. The psychomotor intervention mediated by creative dance induced large beneficial effects in global motor coordination, rhythm, and balance. These findings suggest that this practice is beneficial for promoting community-dwelling older adults’ healthy aging.

**Trial registration:**

ClinicalTrials.gov Identifier: NCT04311931. Date of registration: March 17, 2020.

## Background

The worldwide demographic older population has gradually increased in the last century, and Portugal follows this tendency [1, 2]. However, older adults' extra years of life expectancy are often unhealthy. According to the literature [3, 4], one in three older adults (+ 60 years) is a person with a disability, whose prevalence increases with age.

The aging process involves bio-physiological changes that, over time, result in the decline of the individual's fitness, and skills resulting in disability [5, 6]. Multiple studies demonstrate that psychomotor parameters depending on the energy system and joint mobility, such as aerobic endurance, strength, and flexibility, are crucial for maintaining independence in daily life activities through aging [7, 8]. However, some psychomotor parameters heavily rely on information processing and integration are also essential for maintaining independence, such as coordination abilities and balance [7–9].

Coordination is extremely relevant to the easy and fluid performance of activities of daily living or other more demanding ones [10, 11]. Coordination may be defined as “*the patterning of body and limb motions relative to the patterning of environmental objects and events*” [7]. Neurophysiological mechanisms adjacent to this ability regulate the muscle action synchronization to perform more or less complex movements involving gross or fine motor skills [12]. An important skill intrinsically associated with coordination is rhythm, as the results of these abilities are related, recruit the same brain areas, and utilize identical information processing mechanisms [13, 14], suggesting that they are abilities that integrate the same construct. Rhythm implies the temporal organization of movements by involving timing and synchronization [15, 16]. Global motor coordination and rhythm depend on sensorial perception, body awareness, body schema, postural control, temporal and spatial organization, perception of affordances, processing speed, attention, memory, and planning [7, 17, 18].

Despite the relevance of motor coordination and rhythm for older adults´ cognitive and physical functioning, health, and quality of life [11, 19], there is a gap in the literature concerning validated tests for assessing community-dwelling older adults' global motor coordination and rhythm. Consequently, there is a lack of studies researching the effects of psychomotor, exercise, or other interventions targeting older adults on these parameters.

Balance is defined as “*the process by which we control the body´s center of mass, with respect to the base of support, whether is stationary or moving*” [7]. Literature reports a men-women balance decline rate of 2–3% every 5 years after the age of 60 years and reaching 5–10% after the age of 80 years [20, 21]. Balance aging decline is a problem because it has been identified as the main risk factor for falls in older adults [22, 23].

The World Health Organization (WHO) emphasizes that maintaining healthy behaviors throughout life, particularly engaging in regular physical activity, contributes to reducing the risk of diseases, improving physical and mental capacity, and delaying care dependency [6]. Psychomotor interventions use body and movement experiences as mediators and engage older adults in movement activity while stimulating cognitive, physical, and affective-emotional competencies [24, 25].

Expressive modalities are some psychomotor intervention practices [26]. In this context, creative dance is a pertinent modality for mediate psychomotor intervention with older adults [24] since it is a pleasant practice that can induce benefits in older adults' physical function, physical health, and cognition [27, 28]. This practice integrated movement created by the participants, intending self-expression and communication [27, 29, 30]. To achieve this, participants are encouraged to explore a variety of movement solutions, rather than relying on a single correct answer [30, 31]. Thus, this practice privileges improvisation, composition, and temporality, allowing the stimulation of coordination and rhythm abilities, among other competencies [26, 31].

However, although several studies emphasize the importance of rhythmic movement, dance, and other similar interventions for older people [10, 18, 32, 33], no studies were found determining the effects of dance on their global motor coordination and rhythm. On the other side, dance interventions have been identified as contributing to a decrease in the risk of falls, by inducing improvements in balance [34, 35]. However, the results showing dance benefits on balance in healthy older adults – with the absence of cognitive impairment or disabling disease – are inconclusive due to the small number of studies focused on this theme [28].

Given the above, we have hypothesized that a psychomotor intervention mediated by creative dance would induce improvements in global motor coordination, rhythm, and balance. Thus, the main objective of the present study was to determine the effects of a creative dance intervention on older adults' global motor coordination, rhythm, and balance. For this purpose, a test to assess global motor coordination and a test to assess rhythm in community-dwelling older adults were developed, and their reliability and validity were determined.

## Methods

### Study design and participants

This study includes two phases. In the first phase, two tests were developed and tested aiming at older people: 1) an instrument assessing global motor coordination; and 2) an instrument assessing rhythm. The reliability and validity of both tests were analyzed. In the second phase, a 12-week single-blinded non-randomized clinical trial (NRCT) study was performed following a quasi-experimental design (between March 2020 and July 2020).

For the NRCT study, participants were non-randomly allocated by convenience into the experimental group (EG: attended a psychomotor intervention mediated by creative dance) or the control group (CG: maintained their daily routines). Allocation was performed to ensured that control and experimental groups were matched by age, educational background, and sex, and that experimental group participants had no impediments to attending sessions at the designated weekly schedule. After the study's end, the CG was offered a psychomotor intervention mediated by creative dance. The present study was reported following the SPIRIT 2013 checklist (https://www.spirit-statement.org/publications-downloads/). The protocol was registered at ClinicalTrials.gov (ID: NCT04311931).

Participants were community-dwelling older adults from Portugal recruited through verbal invitations and leaflets addressed to institutions frequented by older people (e.g., senior universities, and recreational centers). The inclusion criteria were the following: a) aged 60 years or older; b) absence of cognitive impairment assessed by the Clock Drawing Test (CDT) (score > 18 points) [36]; c) living independently in the community; d) absence of physical disabilities compromising the creative dance intervention participation; e) and not attending any creative dance intervention in the past 12 months. Three of the forty-one eligible volunteers for this study were excluded. Therefore, 38 participants (60–84 years old) were allocated into the EG (female: 17; male: 2) and CG (female: 18; male: 1). Of these, 34 completed the study, and four were lost to follow-up. Intention-to-treat analysis was performed, and accordingly, all 38 participants were included in the data analysis.

The development and validation of the global motor coordination and rhythm test tasks were performed before the NRCT study (before the group allocation). The sample included the first 30 NRCT participants (3 men and 27 women) who met the abovementioned inclusion criteria. The sample size was determined using the ratio of the number of subjects (N) to the number of items from each test/subtest (p), ensuring a minimum value ranging from three to 10 [37, 38]. This sample characteristics and results did not differ from the NRCT sample.

**Fig. 1:**
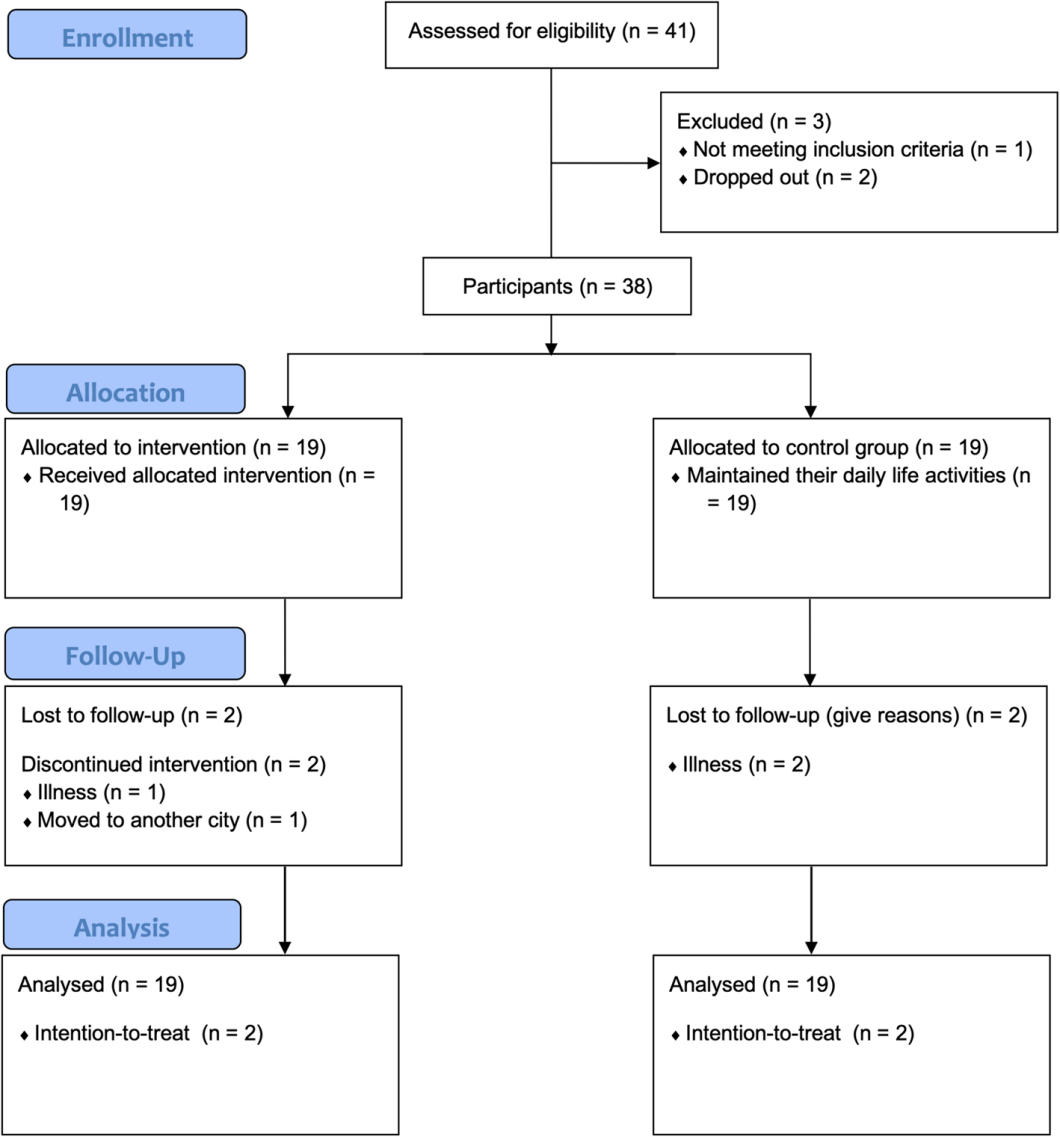
Flow diagram

All participants gave their informed consent before participation. This study followed the Declaration of Helsinki ethical guidelines and was approved by the University of Évora Ethics Committee (CÉ-UÉ), under reference number 16012 (Fig. 1).

### Procedures

Phase 1 (test tasks adaptation and validation): The tasks to assess older adults´ global motor coordination and rhythm were developed following relevant literature reviewing and expertise consultation as recommended [39, 40]. The global coordination test was developed inspired by the Motor Skills Assessment Test (MSAT) for children aged 6 to 8 years [41, 42]. For that, the procedures of the original Laterality test were modified and adapted to target older adults. The tasks to assess older adults´ rhythm were developed inspired by the rhythm neuro-psychomotor function that is included in the Battery for neuro-developmental psychomotor functions (NP-MOT) [16], and on the global praxis factor that is included in the Psychomotor Battery (PMB) [43], besides considering the framework supporting the Montreal Battery for the Evaluation of Amusia [44]. The tasks are explained in the outcome measures section.

After these procedures, a test–retest reliability was performed similarly to the method followed by Almeida et al., [17] study and in line with the guidelines outlined by Shrout & Fleiss [45]. Two fixed and trained evaluators with degrees in Human Kinetic Sciences conducted the test–retest design in a controlled setting to evaluate reliability. The test procedures were standardized to minimize bias or data collection errors. For the intra-rater reliability assessment, one evaluator measured the same participant twice, with a week between each measurement. In the first evaluation, participants performed a practice trial to minimize learning effects and ensure understanding of the test, while only the evaluation trial was conducted in the second. Following this, the inter-rater reliability assessment was carried out by two independent evaluators, measuring the same participant twice. Participants’ instructions were randomly alternated across the evaluators to control potential bias arising from differences in instruction.

Phase 2 (NRCT): Assessments were performed at baseline and post-intervention (after 12 weeks). At baseline, participants completed a practice trial before the evaluation trial to reduce learning effects and ensure understanding of the tests. At post-intervention, only the evaluation trial was conducted. Participants were assessed individually by the same trained evaluator, who has a degree in Human Kinetic Sciences. The evaluator was blind to the study’s objectives and group allocation. Participants were assessed in a laboratory under the same controlled environmental conditions (i.e., a silent room with a temperature around 25 degrees, with adequate lighting). The assessments were performed always in the morning (i.e., 9 to 12 am), starting with the questionnaires, followed by body composition and global motor coordination, rhythm, and balance tests. In addition, participants were instructed to ensure adequate rest, wear comfortable clothing, and avoid strenuous physical activity prior to the test days. A 72-h interval was maintained between assessments and the first and last sessions to prevent carry-over effects and fatigue.

### Outcome measures

#### Global motor coordination

**Fig. 2:**
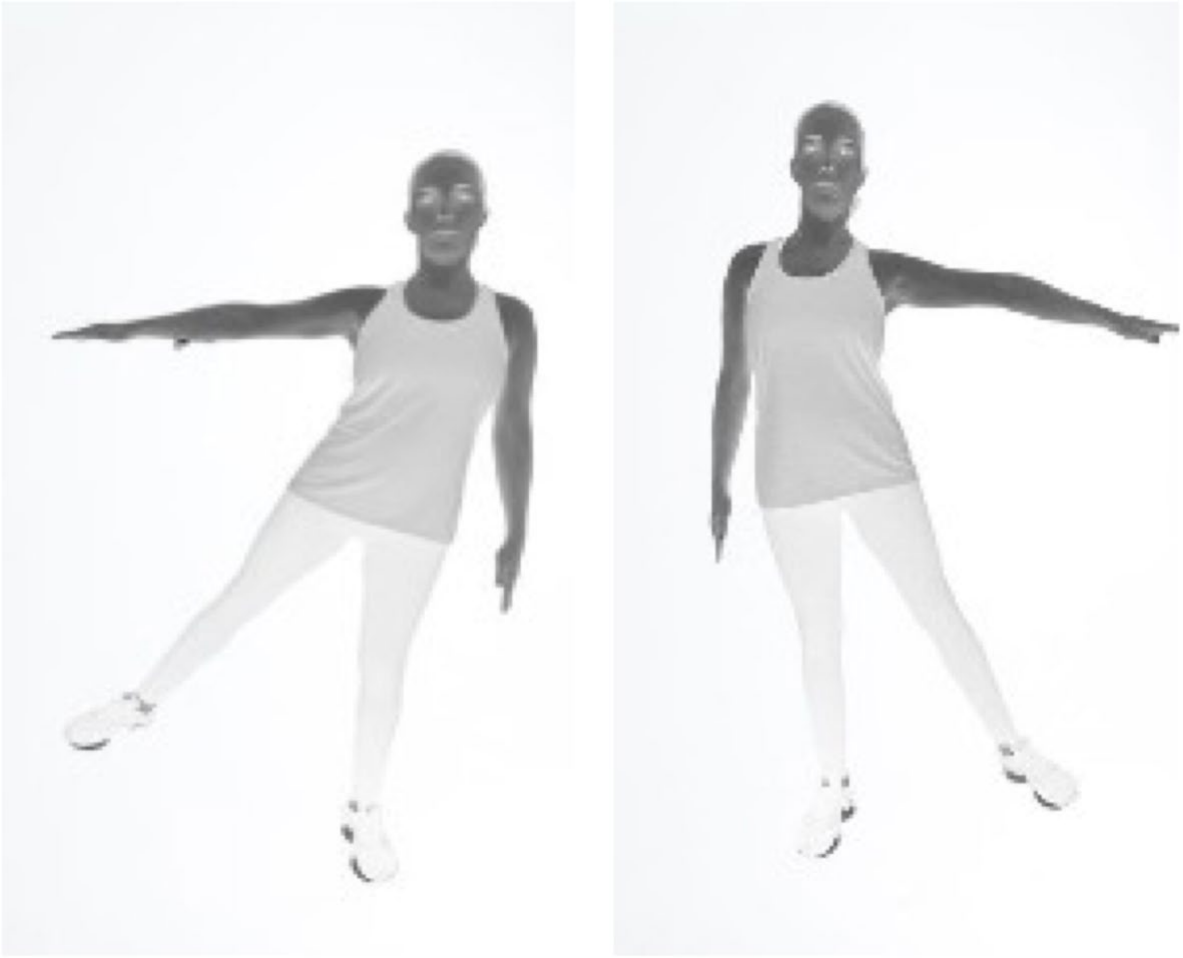
Association and dissociation of movements tasks

Global motor coordination assessment included three tasks, which were developed inspired by the Laterality test from the MSAT [41, 42]. The original MSAT three tasks assess the association and dissociation of movements, after a verbal explanation given to the individual: task i) to separate and join the leg and arm on the same side simultaneously, first the right side (four times) and then the left side (four times) (Fig. 2); task ii) to separate and join the leg and arm on the same side simultaneously, switching sides: first the right side and then the left side, again the right side followed by the left side (repeat that sequence four times); and task iii) to separate the right arm and left leg simultaneously, keeping the left arm and right leg still; repeat the same in opposite (repeat that sequence four times). For the test adaptation to the older people, the tasks were the same but were performed consecutively four times after the rater provided a verbal explanation and demonstration. Each task ranges from 1 (worst) to 4 (best) points. Task scoring is assigned as follows: 1 point if the individual is unable to perform any of the required task movements; 2 points for an out-of-sequence, wavering, or inaccurate performance, including errors such as incorrect limb adduction or abduction; 3 points for a performance that demonstrates minor insecurity but is mostly accurate; and 4 points for an accurate and confident performance. The total score for the global motor coordination variable ranged from 3 (worst) to 12 (best) points. The test takes approximately 5 min to complete.

#### Rhythm

The rhythm was assessed through the performance of three tasks developed inspired by the auditory-visual-kinesthetic and auditory-perceptual-motor tasks from the NP-MOT [16], on the dissociation and coordination (superiors and inferiors’ limbs) tasks included in PMB [43], and on the framework supporting the Montreal Battery for the Evaluation of Amusia [44], as explained above. These tasks involve rhythmic tapping hands on the legs, clapping hands, or walking. All the tasks were synchronized with the beat set by a metronome.

Thus, the rhythm variables concern the results of the following three tasks: Task i) The rhythm-tapping task involves auditory-visual-kinesthetic perception, upper and lower limb dissociation, and coordination. The task is performed sequentially to the metronome's rhythm at 60 bpm and following tapping structures of the feet and hands, such as Right hand (RH), Left hand (LH), Right foot (RF), Left foot (LF), and pause (p). After a verbal explanation and a practical trial (at baseline) the evaluator performed the 1st rhythmic structure, which the participant immediately reproduced. This process continued sequentially, with the evaluator demonstrating and the participant reproducing each subsequent rhythmic structure, up to the 6th structure. The six task structures are the following:

- *Hands structures*
  - 1st structure: 4RH p 3RH p 4RH
  - 2nd structure: 4LH p 3RH p 3RH
- *Feet structures*
  - 3rd structure: 3RF p 2LF p 2LF
  - 4th structure: 3LF p 2RF p 2RF
- *Hands and feet structures*
  - 5th structure: 4RH p 2LF p 2LF
  - 6th structure: 3LH p 3RF p 3RF.

Each rhythmic structure is scored as 1 point when successfully performed (carries out the task by reproducing the structure and being synchronized with the metronome rhythm set at 60 bpm) and 0 points when performed incorrectly. The overall score is the sum of the points obtained in the hands phrase, the feet phrase, and the hands and feet phrase, ranging from 0 (worst result) to 6 points (best result).

Task ii) The rhythm-clapping task concerns an auditory-perceptual-motor task that only involves clapping in rhythm synchronized with a metronome at 90 bpm, 60 bpm, and 120 bpm. Task iii) The rhythm-walking concerns an auditory-perceptual-motor task that only involves walking in rhythm synchronized with a metronome at 90 bpm, 60 bpm, and 120 bpm. For the assessment task, the examiner starts the stopwatch after a few seconds of the metronome starting (4 to 5 beats of the metronome). The maximum duration of the test is 30 s, but the subject may be stopped after 15 s. Each task ranges from 0 (worst) to 6 (best) points (0 points: performance wrong or with an acceleration or deceleration; 1 point: synchronization during ≥ 6 s; 2 points: synchronization during ≥ 15 s). The scoring applies to each cadence. The total rhythm score (rhythm-tapping + rhythm-clapping + rhythm walking) ranges from 0 (worst) to 18 (best) points. The test takes approximately 10 min to complete.

#### Multidimensional balance

Multidimensional balance was assessed by the Fullerton Advanced Balance (FAB) scale [46]. This battery comprises 10 tests, each ranging from 0 to 4 points (higher values indicate better performance). The total score ranges from 0 to 40 points. This test is easy to apply and reliable for older adults (test–retest reliability of 0.96, intra- and inter-rater reliability of 0.91–1.00) [47]. This battery takes 10 to 12 min to complete.

#### Complementary outcomes measures

The sociodemographic characteristics were assessed by a questionnaire by means of an interview based on a script. Cognitive status was evaluated using the CDT [36], which is considered an accurate instrument for identifying cognitive impairments in healthy individuals [48]. The total score ranges from 0 (worst) to 20 (best) points. Following the methodology proposed by Mendez and colleagues (1992) [36], the CDT was administered using a blank sheet of white paper, on which participants were asked to draw the time as ten after eleven. Participants’ body weight (kg) and height (m) were measured with a calibrated device (Seca 760, Hamburg, Germany) and a stadiometer (Seca 206, Hamburg, Germany). Body mass index (BMI) was calculated using the formula (kg/m^2^).

#### Intervention

The EG attended a psychomotor intervention mediated by creative dance following the WHO's recommendations regarding the frequency and duration of physical activity [49] (12 weeks; 3x/week; 60 min; 36 sessions). The creative dance sessions were planned and conducted by the study's second author (PM), who has a degree in dance and psychomotricity. The intervention objectives targeted to explore body awareness (body parts), space (formations, directions, levels, trajectories), time (slow/fast/pause), dynamics (strong/weak/straight/wavy), and interrelationships (pairs and in groups) mediated by preferred and distinctive music styles [30]. The difficulty level of the activities (e.g., movements/velocity) performed was progressively increased throughout the intervention.

The sessions were structured as follows (first 10 sessions: emphasis on body parts/movements; 11–20 sessions: focusing on rhythm and its elements; 21–36 sessions: emphasis on creativity and improvisation): initial dialog (5 min); global activation (10 min); main phase (20 min); choreographic phase (10 min); cool down (10 min), and final dialog (5 min). In the initial dialog, participants recalled what they performed in the previous session and were informed about what would happen in the current session. The global activation phase aimed to promote physiological mobilization through body awareness tasks. In the main phase, participants were encouraged to create their movements (without reproducing an observed movement), expressing their ideas and feelings freely. In this phase, objects such as scarves, balls, and chairs were used as facilitators to stimulate global motor coordination (e.g., bilateral, and asymmetric activities of body parts), rhythm (e.g., different beats, bars, and pauses), and balance (static and dynamic) skills. Concerning the choreographic phase, participants were encouraged to remember the movements performed in the previous session and to create new movements, which were then chosen by the group to be added to a final choreography. In the cool-down phase, participants were encouraged to perform slower movements. In addition, activities such as breathing observation, stretching, and relaxation were used. Finally, in the dialog phase, participants were encouraged to verbalize what they experienced during the respective session. Music with varying rhythms and tempos was used as a mediator in all sessions, carefully selected according to the phase of each session and its specific objectives. Several musical styles were used, such as classical, instrumental, American music, Brazilian popular music, samba, and traditional Portuguese music.

#### Statistical analysis

All data were analyzed using Statistical Package for Social Sciences (SPSS) software, version 28.0. The value established for the level of significance was *p* < 0.05. The test–retest reliability analysis was performed following the Almeida et al., [17] methodology in line with the guidelines outlined by Shrout & Fleiss [45]. Relative reliability was determined using the intraclass correlation coefficient (ICC) [45], with absolute agreement. The ICC values were classified following the guidelines proposed by Koo and Li [50] (< 0.5: poor; 0.5–0.75: moderate; 0.75–0.9: good; and > 0.90: excellent). Absolute reliability was analyzed using the standard error of measurement (SEM = SD √(1-ICC)) and the coefficient of variation (CV = (SD /Mean) *100) [51], both calculated in Excel. To analyze the internal consistency (IC) Cronbach’s alpha was used, with values above 0.9 representing a very good IC [40, 52]. The Spearman correlation coefficient test was used to analyze the criterion and construct validity.

Criterion validity was analyzed by examining the relationship between global motor coordination and rhythmic variables, as the literature [12–14] suggests that these abilities are components of the same construct. Additionally, to our knowledge, no other instruments have been identified that assess these psychomotor parameters in community-dwelling older adults. Construct validity was analyzed by testing the relationship of global motor coordination and rhythmic variables with age, educational level, and cognitive status [53], since literature sustains that they are relied variables that potentially differentiate older persons according the presumed differences in the construct of interest [7, 18, 44]. The magnitude of the correlations was rated as trivial (< 0.1), small (≥ 0.1 and ≤ 0.29), moderate (> 0.29 and ≤ 0.49), large (> 0.49 and ≤ 0.69), very large (> 0.69 and ≤ 0.89) or nearly perfect (> 0.9 and ≤ 0.99) [54].

Missing data analysis was performed using the MCAR test, and they were found to be random, without any manifestation of a trend. An intention of treatment was performed, and the lost data were replaced using the medium series method. Descriptive statistics (mean and standard deviation) were calculated for each result. The variation value between the baseline and post-intervention and between groups was calculated as Delta _within-group_: moment_x_ − moment_x−1_ and Delta _between-group_: EG – CG. Exploratory data analysis revealed that most of the variables did not meet the assumption of normality by using the Shapiro–Wilk test. Thus, the non-parametric statistical analysis was used to perform comparisons. Wilcoxon test was used for within-group comparisons, and the Mann–Whitney test was used for between-group comparisons. Clinical significance concerning the treatment effect was calculated for between-group comparisons, according to the instructions for nonparametric tests [55]. The effect size was computed as r = (Z/√N), and the thresholds of small (< 0.3), medium (0.3–0.8), and large (> 0.8) were considered for this purpose [56].

## Results

The EG and CG had similar characteristics in age (EG: 73.1 ± 5.7 years vs. CG: 75.0 ± 7.2 years), educational level (EG: 6.3 ± 2.8 years vs. CG: 5.5 ± 3.5 years), cognitive status (EG: 19.2 ± 0.4 points vs. CG: 19.3 ± 0.5 points), and IMC (EG: 28.3 ± 3.5 years vs. CG: 27.8 ± 3.3 years). The participants’ attendance in the 36 sessions was 91.4%. The intervention was considered safe by the participants.

Table 1 presents the reliability results. The intra and inter-rater reliability (ICC_2,k_) was excellent, both ranging from 0.962 to 1.00. Cronbach’s alpha indicated a very good IC, with values ranging from 0.961 to 1.00. The F test showed an absence of systematic bias. Regarding criterion validity, significant and positive correlations were found between global motor coordination and rhythm-tapping (r: 0.444, *p* = 0.005), rhythm walking (r: 0.374, *p* = 0.021), and total rhythm score (r: 0.408, *p* = 0.011), showing a moderate magnitude of correlation. In what concerns construct validity, results evidenced positive relationships between rhythm-tapping and educational level (r: 0.330, *p* = 0.043), rhythm-clapping and educational level (r: 0.339, *p* = 0.038), rhythm-clapping and cognitive status (r: 0.351, *p* = 0.031), rhythm-walking and educational level (r: 0.354, *p* = 0.029), rhythm-walking and cognitive status (r: 0.356, *p* = 0.028), and total rhythm score and educational level (r: 0.413, *p* = 0.010). The magnitude of these correlations was moderate.

**Table 1:** Relative and absolute intra- and inter-rater reliability (*n* = 30) for the global motor coordination and rhythm variables

**Outcomes**		**Mean ± SD**	**Relative reliability**	**Absolute reliability**		***F* test**	
			**ICC**_**2.k**_	**SEM**	**CV**	***F***	***p***
**Intra-rater**							
** GMC**							
GMC_Subtest1 (n)	test	3.8 ± 0.5	1.00	± 0.00	± 11.93	1.00	0.50
	retest	3.8 ± 0.5					
GMC_Subtest2 (n)	test	3.6 ± 0.7	0.962	± 0.72	± 18.87	1.00	0.50
	retest	3.6 ± 0.7					
GMC_Subtest3 (n)	test	2.9 ± 0.9	0.977	± 0.71	± 29.76	1.00	0.50
	retest	2.9 ± 0.9					
** Rhythm**							
Rhythm-tapping (n)	test	2.5 ± 2.4	0.996	± 0.82	± 94.81	0.92	0.58
	retest	2.6 ± 2.5					
Rhythm-clapping (n)	test	4.5 ± 1.8	0.992	± 0.85	± 39.41	0.92	0.59
	retest	4.5 ± 1.8					
Rhythm-walking (n)	test	4.6 ± 1.6	0.997	± 0.49	± 35.32	1.00	0.50
	retest	4.6 ± 1.6					
**Inter-rater**							
** GMC**							
GMC_Subtest1 (n)	rater 1	3.8 ± 0.5	1.00	± 0.00	± 11.93	1.00	0.50
	rater 2	3.8 ± 0.5					
GMC_Subtest2 (n)	rater 1	3.6 ± 0.7	0.962	± 0.72	± 18.87	1.00	0.50
	rater 2	3.6 ± 0.7					
GMC_Subtest3 (n)	rater 1	2.9 ± 0.9	1.00	± 0.00	± 29.76	1.00	0.50
	rater 2	2.9 ± 0.9					
** Rhythm**							
Rhythm-tapping (n)	rater 1	2.5 ± 2.4	0.993	± 1.09	± 93.82	0.92	0.59
	rater 2	2.7 ± 2.5					
Rhythm-clapping (n)	rater 1	4.5 ± 1.8	0.995	± 0.67	± 38.49	1.02	0.48
	rater 2	4.5 ± 1.7					
Rhythm-walking (n)	rater 1	4.6 ± 1.6	0.988	± 0.97	± 36.43	0.93	0.58
	rater 2	4.5 ± 1.7					

*SD* standard deviation, *ICC* intraclass correlation coefficient, *SEM* standard error of measurement, *CV* coefficient of variation, *GMC* global motor coordination

Table 2 displays the analyses within and between groups in the psychomotor parameters and multidimensional balance variables. Within-group comparisons evidenced that the EG showed enhancements from baseline to post-intervention in all variables, namely: global motor coordination (Δ_within-group_: 2.9 ± ; *p* < 0.001), rhythm-tapping (Δ_within-group_: 2.2; *p* < 0.001), a rhythm-clapping (Δ_within-group_: 1.8; *p* < 0.001), rhythm-walking (Δ_within-group_: 1.7; *p* < 0.001), total rhythm score (Δ_within-group_: 5.7; *p* < 0.001), and multidimensional balance (Δ_within-group_: 3.5; *p* < 0.001). For the CG, only the variable rhythm-tapping showed significant improvements from baseline to post-intervention.

**Table 2:** Effects of the psychomotor intervention mediated by creative dance in psychomotor parameters and multidimensional balance. EG (*n* = 19) and CG (*n* = 19)

	**Baseline** **Mean ± SD**	**Post-intervention** **Mean ± SD**	***p-value***
Global motor coordination (3–12 points)			
EG	8.0 ± 1.5^a^	10.9 ± 0.9^a^	< 0.001
CG	9.2 ± 1.9	9.1 ± 1.7	0.662
Rhythm-tapping (points) (0–6 points)			
EG	3.0 ± 1.6	5.2 ± 1.1^a^	< 0.001
CG	2.9 ± 2.1	3.9 ± 1.9	0.002
Rhythm-clapping (0–6 points)			
EG	3.4 ± 1.4	5.2 ± 1.1^a^	< 0.001
CG	4.0 ± 1.9	3.5 ± 1.7	0.088
Rhythm-walking (0–6 points)			
EG	3.2 ± 1.5	4.9 ± 1.3^a^	< 0.001
CG	4.2 ± 1.8	3.8 ± 1.7	0.104
Total rhythm score (0–18 points)			
EG	9.6 ± 3.5	15.3 ± 3.1^a^	< 0.001
CG	11.2 ± 4.9	11.2 ± 4.8	0.830
Multidimensional balance (0–40 points)			
EG	31.7 ± 3.1	35.2 ± 2.5^a^	< 0.001
CG	30.1 ± 4.5	29.4 ± 5.5	0.193

*SD* standard deviation

^a^between-group comparisons, *p* < 0.05

The descriptive analysis revealed that men in both the EG and CG demonstrated results similar to those of the women. Male participants in the EG showed improvements across all variables, reaching levels comparable to the female participants and the overall EG (Δ_within-group global motor coordination_: 3.0; Δ_within-group rhythm-tapping_: 3.0; Δ_within-group rhythm-clapping_: 2.5; Δ_within-group rhythm-walking_: 1.0; Δ_within-group total rhythm score_: 6.5; Δ_within-group multidimensional balance_: 2.0). On the other hand, the male participant in the control group maintained his baseline performance.

**Fig. 3:**
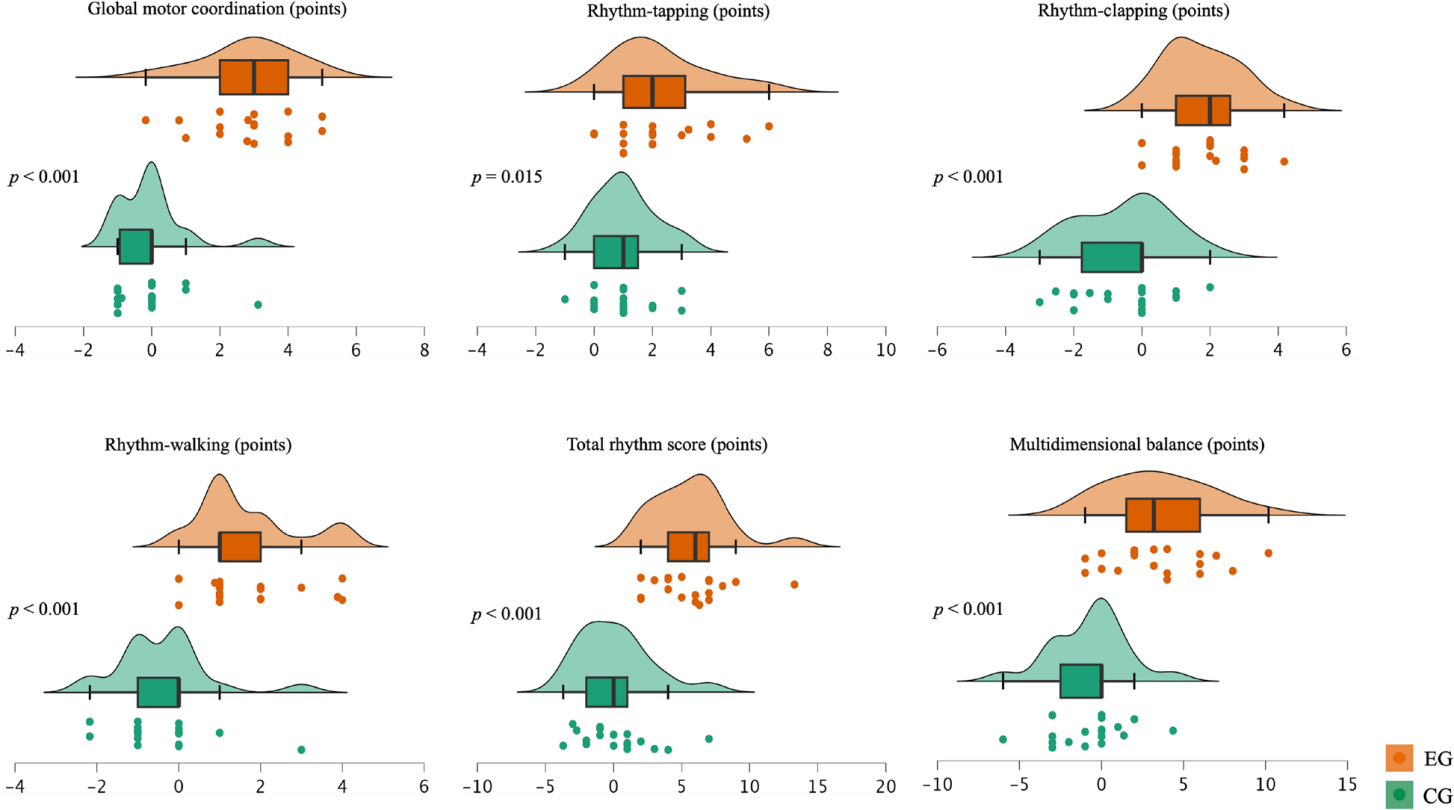
Jitter, box, and density plots comparisons between groups concerning pre-post-intervention delta. Median values are represented by bold vertical lines, and the first and third quartiles are indicated by the lower and upper hinges, respectively. Significant differences between EG and CG, *p* < 0.05. EG (*n* = 19) and CG (*n* = 19)

Concerning the between-group comparisons, no significant differences were found at baseline, except for the variable global motor coordination. In addition, the descriptive analysis revealed that the three men in both the EG and CG demonstrated baseline results similar to those of the women and the overall participants. At post-intervention, the between-group comparison showed significant differences in all variables analyzed, *p* < 0.05. The comparisons between groups concerning pre-post-intervention delta (Fig. 3) also evidenced significant differences in all studied variables, *p* < 0.05. The effect size calculated considering delta results for EG and CG was large for global motor coordination (0.73), ranged from medium to large for the rhythm variables (0.40 – 0.74), and was large for multidimensional balance (0.61).

## Discussion

Concerning the findings of the study phase 1 (tests adaptation and validation), our study results showed that the developed global motor coordination and rhythm tests were reliable and valid in community dwellings populations. Specifically, the tests were shown to be valid for women, with results strongly supporting their use in this group. Regarding men, although the findings were consistent with those observed for women, the reduced number of male participants implies that the validity of the tests for men can only be extrapolated and should be confirmed in future studies.

To the best of our knowledge, there are no other instruments assessing these psychomotor parameters in older people. For all intra and inter-rater analyses, relative and absolute reliability tests showed excellent correlations and small standard errors between measurements [50], as well as a very good IC [52]. These findings evidenced that both tests are stable over time and between raters. The fact that the known tests assessing global motor coordination and rhythm were aimed at children makes comparison with our study results difficult in what concerns its reliability in older adults. Nevertheless, compared to our study, the intra-rater reliability of the rhythm test designed for children (NP-MOT test) presented lower values (0.78 to 0.88) [16]. This difference may be related to the sample used by the Vaivre-Douret study [16], i.e., intra and inter-variability in the children's stages of acquisition and development skills (e.g., body awareness, and motor and coordination skills), compared to older adults.

Regarding criterion validation, as we did not find other instruments assessing global motor coordination and rhythm in community-dwelling older adults, we opted to analyze the relationships between rhythm and our global motor coordination tests as both depend on similar sensory perceptual and processing mechanisms [7, 14, 17, 18]. These results demonstrated a moderate magnitude of correlation, reinforcing the experts' opinions and providing confidence that the instruments effectively assess the psychomotor parameters they are designed to evaluate, as well as supporting the assumption that these abilities are part of the same construct.

In what concerns construct validity, it was observed that the rhythm tasks results were positively associated with the construct of interest variables [7, 18, 44] with a moderate magnitude, such as educational level and cognitive status, although there were not found significant correlation between these variables and age. No significant correlations were found between these constructs of interest variables and global motor coordination. Nonetheless, rhythm and global motor coordination tests were able to discriminate changes induced by the psychomotor intervention using creative dance. In summary, both tests were feasible, as all study participants were able to perform the tasks with greater or lesser success. Considering that motor coordination and rhythm are fundamental parameters for older adults' functioning, health, and quality of life [11, 19], these tests could serve as new valuable clinical tools. They are quick and easy to administer, cost-free, and suitable for use with community-dwelling older adults.

Regarding the study phase 2 findings, our results suggested the effectiveness of the psychomotor intervention mediated by creative dance by inducing improvements in global motor coordination, rhythm, and balance. These improvements observed in the EG were clinically relevant, as they all had a large magnitude of effect, except for the rhythm-tapping result, which was medium. Globally, the CG participants maintained their results over the intervention period. The only exception was rhythm-tapping. Few studies have used a psychomotor intervention mediated by dance in older adults, such as the studies of [24, 25]. However, these studies used a different dance genre (dance therapy) and samples (institutionalized older adults with dementia and adults with high support needs, respectively) which makes the comparison with our study difficult. In this way, we only compared our study results with other dance or creative dance interventions [30, 31].

Overall, our psychomotor intervention mediated by creative dance did not show adverse effects, revealing to be safe and viable for community dwellings. This observation aligns with Clifford and colleagues' systematic review and meta-analysis study [27]. The attendance (91.4%) and dropout rates of the present study were also in line with the previous study (most studies showed an attendance rate of at least 80%). The fact that creative dance promotes creative and spontaneous movements, music, and enjoyable and motivating activities embedded in a social environment [57] may explain the high attendance rate and the lack of dropouts.

Specifically, concerning the present study's global motor coordination and rhythm results, more studies focusing on coordination in older adults are needed, which restricts our discussion. However, our study's improvements in coordination and rhythm may be explained by the reciprocal association between them. It has been demonstrated that rhythmic training enhances coordination and laterality [12]. Both these psychomotor parameters depend on sensory/perceptual, neurocognitive (e.g., inhibitory control), and motor (e.g., motor planning) components working closely for an adaptative and efficient motor control and movement regulation. [7, 10, 34, 58–60]. Also, there is a relation between rhythm and walking, given that older adults with rhythm impairments, particularly poor beat perceivers, tended to take shorter steps when faced with unfamiliar stimuli [18]. This coordination/rhythm bidirectional relationship evidenced the importance of body-oriented interventions, such as dance, using the body as the principal vehicle for expression [25]. Moreover, integrating coordination activities into creative dance interventions is important since they positively affect neuroplasticity due to neurocognitive demands [57].

Interestingly, rhythmic training in healthy older adults has been associated with other parameters improvement, particularly balance [19], which is in line with our study results. Other studies using creative dance interventions such the study of Cruz-Ferreira [30] or the study of Joung and Lee [31] also reported balance enhancements at post-intervention, showing treatment effects of 0.57 and ranging from 0.21 to 0.39, respectively. However, compared to the present study, the previous studies had different durations and frequencies of interventions (8 or 24 weeks; 2 or 3 days/week), different outcome measures to assess balance (Timed Up and Go test; Berg Balance Scale). In addition, these studies used different methods to calculate the magnitude of the treatment effect, whereby the comparison should be interpreted with caution. Nevertheless, the interventions performed by the previous studies were similar to those of our study, which helped explain the balance improvements. Likewise, our intervention engaged participants in postural control constraints, multidirectional variations, spatiotemporal dynamics, or different external stimuli supported by music, promoting dynamic balance training and neuromotor changes [30, 31]. Additionally, the current psychomotor intervention emphasized bilateral and asymmetric activities of body parts to enhance coordination. Tasks were performed at different beats, bars, and pauses to stimulate rhythms, which may have also contributed to improved balance [19]. It should be noted that balance improvements can also help prevent falls and fall-related injuries, which can positively impact older adults' quality of life [35].

Thus, our study results suggest the positive effects of psychomotor intervention mediated by dance to promote global motor coordination, rhythm, and balance in community dwellings. The fact that creative dance is not a traditional type of exercise program with repetitive and mechanized movements or repetitions may encourage older adults to participate. Besides, this practice promotes the social environment and uses low-cost materials. Suggestions for future research include cessation training (follow-up) since it may also be relevant to determine if the improvements are maintained or reversed.

The present study also has strengths and limitations. Among the strengths, we highlight the innovative nature of the intervention, as well as the development and validation of the tests measuring global motor coordination and rhythm. Another strength was the higher intervention attendance rate. One limitation concerns the criterion and construct validity of the developed tests, as the magnitude of the tested correlation was moderate. Although the test's internal consistency was excellent, additional studies focusing on this matter are needed, particularly because the rhythm tasks has not been normalized for older people. This issue may introduce bias in the interpretive ranges and affect its accuracy. Other limitations that should be referred to concern the NRCT design and small sample size, which implies some caution in findings the generalization. Finally, women were predominant in the study, and although this is consistent with the participant proportions in other dance interventions [61], additional caution is warranted when generalizing the findings to the broader male population. Further studies are recommended to address this issue.

## Conclusions

The current study supports the reliability and validity of the global motor coordination and rhythm tests in community dwellings, although it recommends confirming these findings, especially for men. Both tests have suggest being valuable clinical assessments for either clinical or non-clinical care professionals. Our study results advocated that the psychomotor intervention mediated by creative dance promoted improved global motor coordination, rhythm, and balance in community-dwelling older people. The improvements were clinically relevant, as almost all had a large magnitude effect. These findings suggest that psychomotor intervention mediated by creative dance promoted benefits in parameters closely related to older people's health, well-being, and independence.

## Data Availability

The datasets used and/or analyzed during the current study are available from the corresponding author upon reasonable request.

## Abbreviations

WHO: World Health Organization
NRCT: Non-randomized clinical trial
EG: Experimental group
CG: Control group
MSAT: Motor Skills Assessment Test
NP-MOT: Battery for neuro-developmental psychomotor functions
PMB: Psychomotor Battery
BMI: Body mass index
ICC: Intraclass correlation coefficient
SEM: Standard error of measurement
IC: Internal consistency
